# Supplementary material for: Real World Patterns of Antimicrobial Use and Microbiology Investigations in Patients with Sepsis outside the Critical Care Unit: Secondary Analysis of Three Nation-Wide Point Prevalence Studies
Source: J Clin Med. 2019 Aug 29;8(9):1337. doi: 10.3390/jcm8091337 (PMC6780948; doi:10.3390/jcm8091337)
Supplement: Supplementary file 1 [file jcm-08-01337-s001.pdf]

Table S1. Subgroup analysis of patient characteristics for patients from emergency departments and general wards.

Variable	Current Ward Type		P-value
	Emergency Department	General Ward	
**Microbiological investigation**			
Blood culture, n (%)	63 (44.37)	358 (42.57)	0.69
Sputum culture, n (%)	16 (12.60)	92 (12.74)	0.96
Urine culture, n (%)	49 (35.00)	272 (32.69)	0.59
Wound culture, n (%)	8 (9.31)	77 (5.71)	0.17
Other culture, n (%)	29 (15.03)	146 (13.79)	0.65
**Antibiotic prescription**			
Prescription present, n (%)	122 (63.21)	653 (61.67)	0.68
Number of antibiotics (median, range)	1 (0-3)	1 (0-5)	0.91
**Sepsis criteria filfilled**			
SIRS	132 (68.39)	607 (57.32)	0.004
SOFA	110 (56.99)	630 (59.49)	0.52
**Management**			
Sepsis screening tool, n (%)	51 (27.27)	170 (16.86)	0.001
Sepsis Six complete, n (%)	41 (21.240	125 (11.82)	<0.001
Senior review, n (%)	11 (5.70)	110 (10.34)	0.07

Table S2. Characteristics of antibiotics prescription and microbiological investigations depending on the infection source.

Infection source	Number of antibiotics, median (range)	Number of investigations, median (range)	Blood culture taken, n (%)
Respiratory	1 (0-5)	1 (0-4)	176 (40.46%)
Urinary	1 (0-4)	1 (0-4)	46 (52.87%)
Gastrointestinal	1 (0-5)	1 (0-4)	81 (39.51%)
CNS	1 (0-4)	1 (0-4)	29 (49.15%)
Endocarditis	2 (0-3)	2 (0-2)	3 (37.50%)
Line infection	2 (0-4)	2 (0-2)	5 (71.43%)

CNS, Central Nervous System
